# Post-Pandemic vs. Pandemic Food Behaviors in a National Sample of Polish Adolescents: The PLACE-19 Study

**DOI:** 10.3390/nu18183029

**Published:** 2026-09-16

**Authors:** Dominika Guzek, Dominika Skolmowska, Dominika Głąbska

**Affiliations:** 1 Department of Food Market and Consumer Research, Institute of Human Nutrition Sciences, Warsaw University of Life Sciences (SGGW-WULS), 159C Nowoursynowska Street, 02-776 Warsaw, Poland; dominika_guzek@sggw.edu.pl; 2 Department of Dietetics, Institute of Human Nutrition Sciences, Warsaw University of Life Sciences (SGGW-WULS), 159C Nowoursynowska Street, 02-776 Warsaw, Poland; dominika_skolmowska@sggw.edu.pl

**Keywords:** food behaviors, Food Behavior Checklist (FBC), fruit and vegetable intake, dairy intake, fat intake, dietary quality indicators, food security, adolescents, PLACE-19 Study

## Abstract

**Background/Objectives**: Food behaviors in the general population and among adolescents during the COVID-19 pandemic have been well documented through numerous comparisons with the pre-pandemic period, but the post-pandemic situation has not yet been sufficiently described. The aim of this study was to compare post-pandemic vs. pandemic food behaviors in a national sample of Polish adolescents within the framework of the PLACE-19 Study. **Methods**: A population of *n* = 1019 secondary school adolescents recruited based on a quota sampling of Polish secondary schools was included in the analysis (*n* = 690 female, *n* = 329 male). Data were gathered using the Computer-Assisted Web Interview (CAWI) technique, and food behaviors were assessed using the Food Behavior Checklist (FBC). Analyses were conducted for the fruit and vegetable quantity, fruit and vegetable variety, sweetened beverage, milk/dairy, healthy fat, diet quality, and food security subscales, as well as questions related to fruit and vegetable and total scale. **Results**: In the total studied population as well as in subgroups of female and male individuals, higher scores were observed in the post-pandemic period than in the pandemic period for the fruit and vegetable quantity subscale (*p* < 0.005), the fruit and vegetable variety subscale (*p* < 0.001), and questions related to fruit and vegetable (*p* < 0.001). At the same time, lower scores in the post-pandemic period than in the pandemic period were observed for the milk/dairy subscale (*p* < 0.05) and the healthy fat subscale (*p* < 0.05). At a one-year interval, 43.7% of the population showed an improvement in their total FBC score, whereas 40.3% showed a worsening, while a significant association was observed for sex (*p* = 0.0114), with females more frequently presenting total FBC score improvement than males (44.9% vs. 41.0%). **Conclusions**: Food behaviors among adolescents changed after the COVID-19 pandemic, which may be related to both internal factors (including the level of experienced stress) and external factors (including exposure to peers). The novel situation observed in the post-pandemic period may warrant further analysis to determine whether these pandemic-associated changes will persist and to identify potential new challenges.

## 1. Introduction

According to the World Health Organization (WHO), adolescents are defined as individuals aged 10–19 years [1], while the U.S. Department of Health and Human Services and the Food and Drug Administration (FDA) define adolescence as the period between 12 and 21 years of age [2]. Adolescence is a critical stage of development, marked by significant physical, psychological, emotional, and social changes [3]. During this phase, individuals increasingly gain autonomy over their food choices, which often leads to suboptimal dietary patterns, even though nutritional status during this period is fundamental for proper physical and psychological development [4]. Importantly, dietary habits formed during adolescence tend to persist into adulthood, potentially contributing to an increased risk of obesity and diet-related diseases in later life [5].

The prevalence of overweight and obesity among adolescents continues to rise globally, and the most recent systematic review and meta-analysis by Zhang et al. [6] found that approximately one in five children and adolescents are affected by excessive body weight. The international Health Behaviour in School-aged Children (HBSC) study [7], based on a 2021/2022 survey conducted across 44 countries and regions, including Poland, confirms this upward trend. In Poland specifically, in the HBSC study, the prevalence of overweight and obesity was high across all age groups, reaching 19% in 11-year-old girls and 41% in 11-year-old boys, 18% in 13-year-old girls and 35% in 13-year-old boys, as well as 13% in 15-year-old girls and 28% in 15-year-old boys [7].

Given this growing public health burden, as indicated by the WHO [8], identifying the determinants contributing to excess weight gain in adolescents has become a research priority, while food behaviors, including food preferences, meal patterns, and eating habits established early in life, may be among the factors most consistently implicated [9]. They are shaped by a complex interplay of genetic and environmental influences [10], and their role may vary depending on the broader societal context, including shifts in eating behavior observed in the post-pandemic period [11].

The COVID-19 pandemic substantially disrupted food behaviors across the general population, as documented in several systematic reviews. In the systematic review of longitudinal studies by González-Monroy et al. [12], the authors reported an increased snacking frequency and an increased preference for sweets and ultra-processed food products, alongside a decreased preference for fruits, vegetables, and fresh foods and reduced overall adherence to healthy diet guidelines. However, the systematic review by Nindenshuti & Caire-Juvera [13] found both healthy and less healthy lifestyle changes, while among 15 studies assessing diet, 13 reported some positive food behavior changes while 9 reported some negative food behavior changes. Similarly, in the systematic review by Mignogna et al. [14], favorable dietary changes were reported, including an increase in the consumption of fruit, vegetables, legumes, cereals, dairy, and olive oil, and a decrease in the consumption of red and processed meat, accompanied by unfavorable changes, namely an increase in the consumption of snacks and sweets and a decrease in the consumption of fish.

Several systematic reviews have also examined food behavior changes specifically in adolescents. In the systematic review by Woods et al. [15], the majority of included studies reported favorable changes, including an increase in the consumption of fruit, vegetables, and grain products, as well as a decrease in the consumption of ultra-processed foods and improved diet quality indices, accompanied by unfavorable changes, including an increase in the consumption of meat, poultry, and eggs, as well as an increased frequency of snacking. On the other hand, in the systematic review and meta-analysis by Na et al. [16], an increase in meal frequency and vegetable intake were reported, and food behavior changes were concluded to be rather positive for adolescents, but mixed for school-age children.

Although food behaviors in the general population and among adolescents during the COVID-19 pandemic have been well documented through comparisons with the pre-pandemic period, considerably less is known about how these behaviors have evolved following the end of the pandemic. Within this area, the number of studies remains limited and, to date, no synthetic analysis has been conducted on a population of Polish adolescents comparing food behaviors in the post-pandemic period with those observed during the pandemic. Therefore, the aim of the present study was to compare post-pandemic vs. pandemic food behaviors in a national sample of Polish adolescents within the framework of the Polish Adolescents’ COVID-19 Experience Study (PLACE-19 Study).

## 2. Materials and Methods

### 2.1. Study Design and Data Collection

The present analysis was based on data collected during the fourth phase of the PLACE-19 Study, carried out between May and June 2021. By that time, nationwide lockdown measures in Poland had been lifted, restrictions had been gradually eased, and school education had returned to pre-pandemic conditions [17]. WHO documents from 2021 already used the term “post-pandemic,” referring to this period as the “post-pandemic period” [18], or the “post-pandemic era” [19]. Moreover, the Partnership for Maternal, Newborn and Child Health (PMNCH), hosted by the WHO, for children and adolescents specifically, introduced the concept of a “post-pandemic world” through a seven-point call to action aimed at directing investment and policy toward the unequal social, economic, and political factors driving the impact of the COVID-19 pandemic and its consequences [20]. Based on this, the data gathered within the study for the period of May–June 2021 were referred to as post-pandemic, and the data from one year earlier (May–June 2020) were referred to as pandemic.

The PLACE-19 Study was designed as a multi-phase research project examining various aspects of adolescents’ experiences during the COVID-19 pandemic. The fourth phase focused on the situation following the prolonged pandemic period. The first phase focused on hygienic and personal protective behaviors [21]; the second phase assessed general dietary behaviors [22]; the third phase examined psychological determinants of food consumption [23]; and the fourth phase investigated detailed food behaviors.

This study was approved by the Ethics Committee of the Central Clinical Hospital of the Ministry of Interior and Administration in Warsaw (No. 2/2021). All of the procedures of the study followed the guidelines of the Declaration of Helsinki and were carried out by the Institute of Human Nutrition Sciences, Warsaw University of Life Sciences (SGGW). Participants and their parents or legal guardians provided informed consent.

### 2.2. Participants

Participants were recruited through secondary schools selected using a quota sampling procedure. The two-phase procedure was applied, including: (1) sampling counties from voivodeships, based on Poland’s administrative division, which yielded 80 counties across 16 voivodeships; and (2) sampling secondary schools from these counties using the national register of secondary schools, which yielded 400 secondary schools. Detailed recruitment procedures are described in a previous publication [24]. This approach was feasible because of the high net enrollment rate (NER) for secondary schools in Poland—89.38% based on Central Statistical Office (CSO) data for December 2019 [25]. Participation was voluntary and anonymous. Secondary school headmasters were responsible for inviting their students, obtaining informed consents from students and their parents or legal guardians, and distributing a link to the electronic questionnaire, administered using the Computer-Assisted Web Interview (CAWI) technique.

The inclusion criteria were: (1) aged 15–20 years; (2) residence in Poland; (3) ability to read and understand Polish; (4) being a student of one of the secondary schools randomly selected for the fourth phase of the PLACE-19 Study; and (5) provision of informed consent, with parental or legal guardians consent required for participants under 18 years of age. The exclusion criteria included: (1) participation in any previous phase of the PLACE-19 Study; and (2) missing or unreliable data in the questionnaire.

Based on the inclusion criteria, *n* = 1027 individuals were included to the 4th phase of the PLACE-19 Study. Subsequently, *n* = 8 individuals were excluded due to missing or unreliable data in the questionnaire subjected to analysis.

The required sample size was calculated from the estimated population of Polish adolescents (*n* = 2,726,492), based on CSO data [26]. Assuming a 95% confidence level, a 5% margin of error, and a population proportion of 50%, the minimum required sample size was 384 participants. Therefore, the final sample of 1019 adolescents met this requirement. The recruited sample comprised *n* = 690 female and *n* = 329 male respondents, resulting in a female predominance that is consistent with previous phases of the PLACE-19 Study [27,28] and has also been reported in other studies [29].

### 2.3. Food Behavior Checklist

Food behaviors were assessed using the Food Behavior Checklist (FBC), a validated 16-item questionnaire developed by Townsend et al. [30]. The criterion validity of the FBC was established through a significant correlation between the fruit and vegetable subscales and serum carotenoid levels [24]. These findings were subsequently confirmed by Murphy et al. [31] and Blackburn et al. [32], with the study by Blackburn et al. [32] also confirming the correlation with dietary recall variables.

Previous analyses conducted in the PLACE-19 Study used the Adolescents’ Food Habits Checklist (AFHC) [22,33], but the FBC was chosen for the present study because in addition to assessing dietary behaviors, it includes an item assessing food security. This choice was based on the assumption that food security may change in the post-pandemic period following an increase in food insecurity during the pandemic, as reported by the Food and Agriculture Organization of the United Nations (FAO), the International Fund for Agricultural Development (IFAD), the United Nations Children’s Fund (UNICEF), the World Food Programme (WFP), and the WHO [34].

The FBC assesses the frequency of specific food-related practices across five conceptual domains: (1) fruit and vegetable consumption (7 items), (2) dairy intake (2 items), (3) fat-related behaviors (2 items), (4) dietary quality indicators (4 items), and (5) food security (1 item). Questions address participants’ usual eating and drinking habits, including how often they typically consume particular foods and beverages. Several items refer specifically to behaviors during the previous week, including citrus fruit consumption, milk intake, and fish consumption [35].

In the PLACE-19 Study, questions about food behaviors addressed participants’ current situation. At the same time, respondents were also asked to report their food behaviors one year earlier, namely during the COVID-19 pandemic, when remote learning was introduced (i.e., retrospective data from one year prior). For this retrospective section, the questions about the previous week were changed to typical week one year before. Retrospective longitudinal designs like this have been widely used in COVID-19-related research. Notable examples of prominent studies conducted in the context of the COVID-19 pandemic and using the retrospective data include the ZOE COVID Study among adults in the United Stated of America and the United Kingdom [36], and the HEBECO Study among adults in the United Kingdom [37]. In the Polish population, other studies besides the PLACE-19 Study have also used retrospective data collection, including the DAY-19 Study conducted among children [38].

Within the FBC, various scales are applied. Items assessing the frequency of food-related behaviors (9 items) are scored on a 4-point Likert scale: “never”, “sometimes”, “often”, and “always”, with higher scores reflecting more desirable behaviors. For the 7 positive items, a regular scale was applied (from 1 to 4), and for the 2 negative items, answers were reverse-coded prior to analysis (from 4 to 1). The food security item with the same 4-point Likert scale (1 item) was also reverse-coded prior to analysis, so that higher scores consistently reflected more favorable conditions (from 4 to 1). Dichotomous behavioral items with yes/no responses (3 items) all represented positive behaviors, so a regular scale was applied (2 for “yes” and 1 for “no”). Items assessing the quantity of food consumed (2 items) used a 7-point ordinal scale (from 1 to 7), ranging from “none”,”0.5 cup”, “1 cup”, “1.5 cups”, “2 cups”, “2.5 cups”, and “≥3 cups”. The question presenting self-description of diet quality (1 item) used a 10-point scale from “excellent” to “poor” (from 10 to 1) [27].

The calculated total FBC score was compared for each individual respondent for the pandemic and post-pandemic period. An increase or decrease of at least 1 point in total score was classified as improvement or worsening, respectively, as there is no empirically established minimal clinically important difference or validated numerical cut-off for categorizing individual changes in the total FBC score.

As no validated Polish version of the FBC exists, it was translated following international guidelines [39,40]. The translation process involved: (1) forward translation from English to Polish by a fluent speaker with nutrition expertise; (2) backward translation to English by a fluent speaker blinded to the original version; (3) expert review by a panel of nutrition professionals to check semantic, idiomatic, experiential, and conceptual equivalence; and (4) pilot testing with adolescents to confirm comprehension and clarity. During the translation and adaptation of the questionnaire, the original household measure volumes were not changed, as the American cup measure (240 mL) [41] is comparable to the typical Polish serving size for liquids (250 mL) [42]. Minor wording adjustments were made based on pilot feedback. In the present sample, the Polish version of the FBC demonstrated good internal consistency (Cronbach’s α = 0.77 for the total scale).

### 2.4. Demographic and Anthropometric Data

The questionnaire collected demographic and anthropometric data: age, sex, weight, height, economic situation, secondary school, and participation in previous phases of the PLACE-19 Study. All variables were self-reported and were used to describe the sample and to check inclusion and exclusion criteria.

### 2.5. Data Analysis

For further analyses, the subscale structure proposed by Suzuki et al. [43] was applied. The FBC items were grouped as follows: fruit and vegetable quantity (Q6 and Q7), fruit and vegetable variety (Q1, Q3, Q8, Q9 and Q14), sweetened beverages (Q2 and Q4), milk/dairy (Q5 and Q10), diet quality (Q13 and Q16), healthy fats (Q11 and Q12), and food security (Q15). In addition, based on the findings of Murphy et al. [31] and Blackburn et al. [32], an additional 7-item subscale assessing overall items associated with fruit and vegetables was included in the analyses and was referred to as questions related to fruit and vegetable (Q1, Q3, Q6, Q7, Q8, Q9, and Q14).

Body Mass Index (BMI) was calculated as body weight (kg) divided by height squared (m^2^). For participants aged 18 years and older, standard BMI cut-offs were applied: underweight (<18.5 kg/m^2^), normal weight (18.5–25.0 kg/m^2^), overweight (25.0–30.0 kg/m^2^), or obese (>30.0 kg/m^2^) [44]. For participants younger than 18 years, BMI was interpreted using age- and sex-specific percentile cut-offs recommended by the WHO: underweight (<5th percentile), normal weight (5th–85th percentile), overweight (85th–95th percentile), and obesity (>95th percentile) [45]. BMI percentiles were determined using Polish age- and sex-specific growth reference values [46] and the corresponding Polish OLAF growth charts [47].

Results were compared between subgroups defined by: (1) sex (female and male); (2) body mass (underweight, normal weight, overweight, and obesity); (3) age (minors and adults), and (4) economic situation (poor/very poor, average, good, and very good).

### 2.6. Statistical Analysis

Normality of data distribution was assessed using the Shapiro–Wilk test. Because the variables were not normally distributed, differences between subgroups were analyzed using the Mann–Whitney U test. Categorical variables were analyzed using the chi^2^ test. Pandemic and post-pandemic comparisons were performed using Wilcoxon signed-rank test or paired-samples *t*-test, while effect sizes were quantified using the rank-biserial correlation (RBC). Following Cohen’s guidelines, the following thresholds for absolute values were applied: 0.10 for a small effect, 0.30 for a moderate effect, and 0.50 for a large effect [48].

Statistical significance was set at *p* ≤ 0.05. All statistical analyses were performed using Statistica version 8.0 (StatSoft Inc., Tulsa, OK, USA) and Jamovi version 2.6.44 (Jamovi Project, Sydney, Australia).

## 3. Results

The general characteristics of the total adolescents studied in the PLACE-19 Study are presented in Table 1. The study sample included 1019 adolescents, roughly two-thirds were female and one-third were male, with a median age of 17 years. Age did not differ significantly between female and male individuals. Most participants had normal body weight. BMI was significantly higher in male than female adult participants, and the same pattern was observed for BMI percentile among participants under 18. However, the distribution of body mass categories did not differ significantly between females and males (*p* = 0.1431).

The comparison of FBC subscale scores for the adolescents studied in the PLACE-19 Study between the post-pandemic and pandemic periods at the one-year interval is presented in Table 2. Higher scores in the post-pandemic period than in the pandemic period were observed for the fruit and vegetable quantity subscale (*p* < 0.001), the fruit and vegetable variety subscale (*p* < 0.001), the sweetened beverage subscale (*p* = 0.011), and the food security item (*p* = 0.002), as well as for questions related to fruit and vegetable (*p* < 0.001) and total scale (*p* = 0.020). At the same time, lower scores in the post-pandemic period than in the pandemic period were observed for the milk/dairy subscale (*p* < 0.001) and the healthy fat subscale (*p* < 0.001). Effect sizes were generally small to moderate, with the largest effect observed for the milk/dairy subscale.

The comparison of FBC subscale scores for the female adolescents studied in the PLACE-19 Study between the post-pandemic and pandemic periods at the one-year interval is presented in Table 3. Higher scores in the post-pandemic period than in the pandemic period were observed for the fruit and vegetable quantity subscale (*p* < 0.001), the fruit and vegetable variety subscale (*p* < 0.001), the sweetened beverage subscale (*p* < 0.001), and the food security item (*p* = 0.029), as well as for questions related to fruit and vegetable (*p* < 0.001) and total scale (*p* = 0.020). At the same time, lower scores in the post-pandemic period than in the pandemic period were observed for the milk/dairy subscale (*p* < 0.001) and the healthy fat subscale (*p* < 0.001). Effect sizes were generally small to moderate, with the largest effect observed for the milk/dairy subscale.

The comparison of FBC subscale scores for the male adolescents studied in the PLACE-19 Study between the post-pandemic and pandemic periods at the one-year interval is presented in Table 4. Higher scores in the post-pandemic period than in the pandemic period were observed for the fruit and vegetable quantity subscale (*p* = 0.004), the fruit and vegetable variety subscale (*p* < 0.001), and the food security item (*p* = 0.029), as well as for questions related to fruit and vegetable (*p* < 0.001). At the same time, lower scores in the post-pandemic period than in the pandemic period were observed for the milk/dairy subscale (*p* = 0.012), the healthy fat subscale (*p* = 0.035), and the diet quality subscale (*p* < 0.001). Effect sizes were generally small to moderate, with the large effects observed for fruit and vegetable variety subscale and questions related to fruit and vegetable.

**Figure 1:**
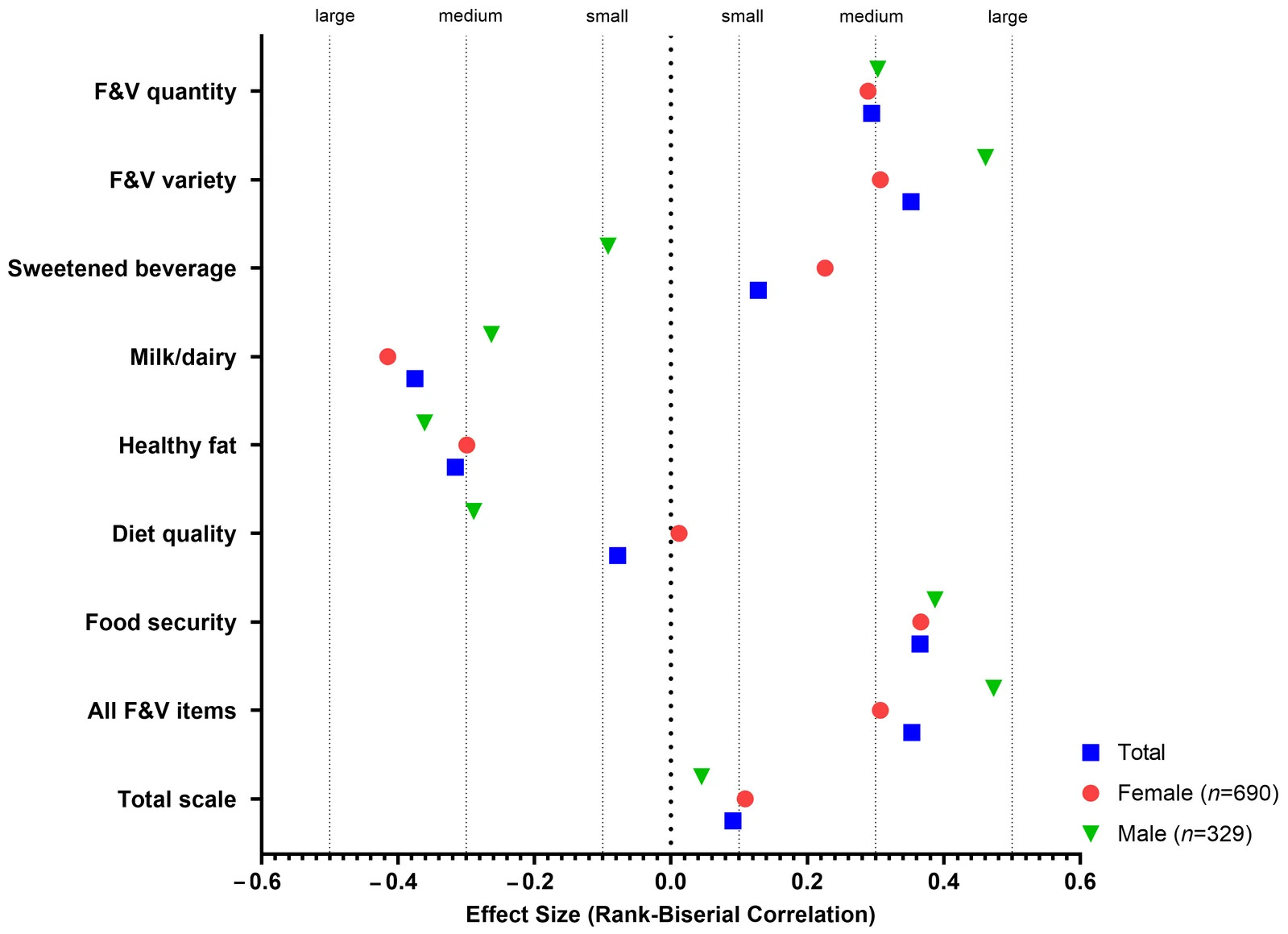
Effect sizes for comparisons of Food Behavior Checklist (FBC) subscale scores for the total, female, and male adolescents studied in the PLACE-19 Study between the post-pandemic and pandemic periods at the one-year interval.

The effect sizes for comparisons of the FBC subscale scores for the total, female, and male adolescents studied in the PLACE-19 Study between the post-pandemic and pandemic periods at the one-year interval are presented in Figure 1. The largest effect sizes were observed for the fruit and vegetable variety subscale, as well as questions related to fruit and vegetable intake for male respondents (effect size > 0.450), while the smallest effect size was observed for the milk/dairy subscale for female respondents (effect size = −0.415).

The distribution of changes in total FBC score for the adolescents studied in the PLACE-19 Study from the pandemic to the post-pandemic period at the one-year interval, stratified by sex, body mass, age, and economic situation, is presented in Table 5. Among the 1019 participants, at the one-year interval, 43.7% presented probable improvement in their total FBC score, while 40.3% presented probable worsening. A significant association was observed for sex (*p* = 0.0114), with females more frequently presenting improvement (44.9% vs. 41.0%) or worsening than males (41.4% vs. 38.0%) and less frequently presenting no change (13.6% vs. 21.0%).

## 4. Discussion

Food choices among adolescents are influenced by a wide variety of factors, indicated by Neumark-Sztainer et al. [49] based on focus-group of adolescents within categories associated with hunger and food cravings, appeal of food, time considerations of adolescents and their parents/guardians, convenience, availability, religion and other cultural aspects, health and other benefits, situation-specific factors, mood, body image, habit, cost, and media. Other authors have indicated other functions of food choices, associated not only with the influence of caregivers but also with need for autonomy and the social function of food among peers [50]. It must be emphasized that adolescents are positioned between their parents and their peers, as both groups shape their ultimate food choices [51]. The influence of parents is the strongest in the case of children, as they create food availability and act as models of eating behaviors; however, this influence becomes weaker for adolescents [52]. At the same time, the scoping review by Chung et al. [53] indicated that peers strongly influence adolescents’ food choices, which is associated with regular contact at school, during breaks, and after classes, while eating behaviors are shaped by perceptions of social norms, creating a specific form of peer pressure [53].

Despite specific determinants of food choices, it must be emphasized that specific food groups may be influenced by various determinants. Namely, the same environment may exert different effects on different food groups because various determinants play dominant roles. This was observed in the study by Loth et al. [54], who compared the determinants of choice for fruit/vegetable intake versus soda and snack foods intake, as contrasting food groups. Similarly, a previous analysis conducted during the second phase of the PLACE-19 Study [55] examined the association between various determinants of food choices (health, mood, convenience, sensory appeal, natural content, price, weight control, familiarity, and ethical concerns) and food product preferences (vegetables, fruits, meat/fish, dairy, snacks, and starches). When comparing food groups, fruit and starch preference scores were positively associated with all food choice determinants, whereas preferences for other food groups were motivated by majority of determinants, but excluding familiarity for vegetables, weight control for dairy, health and ethical concerns for snacks, as well as weight control, familiarity, and ethical concerns for meat/fish [55].

During the COVID-19 pandemic, the typical mechanism described for adolescents in relation to their food behaviors was disrupted. School closures and social isolation reduced direct contact with peers [56] and may therefore have weakened peer pressures contributing to unhealthy food choices. It may be concluded from the pre-pandemic results that peers influence unhealthy food purchases, including the purchase of high-calorie snacks [57].

Moreover, during lockdown, parental supervision of meals may have been stronger than when their children spent most of the day outside the home. French research by Philippe et al. [58] revealed that many parents reported changes in their children’s eating behaviors, parental feeding practices, and motivations for food shopping during this period, including fewer rules, greater use of food for comfort, and more autonomy for children [58]. At the same time, parents appreciated the greater availability of local and fresh foods, more time for food preparation, the opportunity to prepare homemade meals and try new recipes, and the possibility of cooking and eating together as a family [59].

Simultaneously, during the pandemic period, the closure of restaurants and fast-food restaurants also physically removed some food temptations that had previously been available at school or in the surrounding area [60]. In a study of adults from 16 European countries, Molina-Montes et al. [61] reported general decreases in the consumption of fast foods, fried foods, and snacks. Similar observations were also reported for adolescents for fast food [62] and fried foods [33].

The described shift from the mixed influence of peers and parents to the stronger influence of parents may have changed after the pandemic. However, it is possible that this influence was no longer the same as before the pandemic. Some studies revealed that while some adolescents struggled to adapt to socializing in person again post-pandemic, others replaced their previous social behaviors with online interactions [63]. This may have influenced food behaviors, as they are significantly affected by peer socialization.

In the present study, fruit and vegetable quantity as well as fruit and vegetable variety may have improved after the COVID-19 pandemic, while milk/dairy and fat choices may have worsened compared to the pandemic period. To date, only a few small studies can be found comparing food behaviors in the post-pandemic vs. pandemic periods, making the obtained results difficult to compare and discuss in the context of previous research. However, some potential mechanisms may be considered.

On the one hand, potentially worse food behaviors for milk/dairy and fat choices observed post-pandemic compared to the pandemic period may reflect the influential role of peers. A study assessing milk choices among adolescents indicated that the perception of parents’ consumption is an important factor in this age group [64]. However, another study showed positive associations between dairy intake among adolescents and that of their friend groups and their best friends [65]. This may mean that for some products, in the absence of direct peer influence, consumption may have been influenced mainly by parents, but when the influence of peers reappeared, food behaviors may have changed in an unfavorable direction.

On the other hand, potentially better food behaviors for fruit and vegetable quantity as well as fruit and vegetable variety after the COVID-19 pandemic compared to during the pandemic may be associated with reduced pandemic-related stress among adolescents. It is widely known that stress influences food behaviors, as indicated in the systematic review and meta-analysis by Hill et al. [66]. Moreover, it is commonly indicated that stress can change behaviors associated with fruit and vegetables. The Health and Behavior in Teenagers Study (HABITS), a 5-year longitudinal survey tracking a cohort of around 5000 adolescents in Great Britain [67], revealed an association between stress and fruit and vegetable consumption, while a dose–response relationship was indicated, and ss stress increased, the likelihood of unhealthy food behavior also increased. Similarly, the Global School-Based Health Survey (GSHS) [68], based on the data from 61 countries and a population of over 200,000 adolescents, reported that psychological distress was associated with inadequate fruit intake and inadequate vegetable intake. This may mean that when COVID-related stress was reduced, fruit and vegetable intake may have increased.

Interestingly, in the studied population, female individuals more often than male individuals showed improved food behaviors after the COVID-19 pandemic. This difference may be related to traditional gender roles, where women are often expected to take primary responsibility for food preparation [69], alongside external factors motivating girls to eat healthier and maintain a lower body weight to fit traditional norms [70].

In the present study, food security may have improved after the COVID-19 pandemic, being a trend observed in the total sample as well as within both female and male subgroups. Globally, food insecurity emerged among adolescents during the COVID-19 pandemic, driven among others by food insufficiency in low-income settings [71]. In the systematic review by Williams et al. [72] on food insecurity in households with children during the COVID-19 pandemic, most studies assessing this factor reported that the pandemic worsened food security. However, some studies also indicated that despite an initial decrease in food security in September 2020, some decline in food insecurity was observed in May 2021 [73]. This observation corresponds with the results obtained in the present study, as the corresponding periods of May–June 2020 and May–June 2021 were compared and a similar potential increase in food security was indicated during this period. The possible factors contributing to food insecurity during the COVID-19 pandemic include: loss of employment or income resulting in decreased purchasing power [74], increase in food prices, associated with decreased affordability [75], food supply chain disturbances [76], and in the case of children and adolescents, loss of access to school meals [77]. It is likely that the roles of these factors decreased while the study was conducted, as nationwide lockdown measures in Poland had been lifted and school education had returned to pre-pandemic conditions [17]. This may have contributed to increased food security.

The potential role of school in increasing food security should be considered, however, in Poland, a program providing fruits, vegetables, and dairy products is targeted mainly at primary schools rather than secondary schools, which was noted while the study was conducted [78]. Therefore, its direct contribution to improving food security or food behaviors among adolescents attending secondary schools was very limited. Moreover, it should be noted that studies assessing school meals and policies promoting healthy eating in schools in Poland indicate many challenges that should be addressed [79]. This may be particularly relevant when interpreting the observed changes in food behaviors after the reopening of schools, as school-based food provision may have played a greater role among younger children than among secondary school students.

Despite the interesting observations of this study, its limitations must be considered. The most significant issue is that the study relied on a comparison with retrospective data, which entails a risk of recall bias. Moreover, while the studied sample was recruited, a typical non-response bias associated with sampling in public health studies may have occurred owing to the higher proportion of female than male respondents.

## 5. Conclusions

These findings indicate that food behaviors among adolescents changed after the COVID-19 pandemic, which may be related to both internal factors (including the level of experienced stress) and external factors (including exposure to peers). The novel situation observed in the post-pandemic period may warrant further analysis to determine whether these pandemic-associated changes will persist and to identify potential new challenges.

## Figures and Tables

**Table 1:** General characteristics of the total adolescents studied in the PLACE-19 Study (*n* = 1019).

Characteristics	Total (*n* = 1019)		Female (*n* = 690)		Male (*n* = 329)		*p*
	Mean ± SD	Median * (IQR)	Mean ± SD	Median * (IQR)	Mean ± SD	Median * (IQR)	
Age (years)	16.9 ± 1.1	17.0 (2.0)	16.9 ± 1.1	17.0 (2.0)	16.8 ± 1.2	17.0 (2.0)	0.206
BMI (kg/m^2^) (*n* = 327) **	22.2 ± 3.9	21.7 (4.5)	22.0 ± 4.1	21.0 (4.7)	22.5 ± 3.3	22.5 (3.9)	0.021
BMI percentile (*n* = 692) ***	55.1 ± 31.0	58 (58.0)	53.1 ± 31.2	53.5 (57.3)	59.5 ± 30.1	65.0 (53.5)	0.017
	***n*** **(%)**						
Body mass category	**Total (*n* = 1019)**		**Female (*n* = 690)**		**Male (*n* = 329)**		***p***
Underweight (*n* = 72)	72 (7.1%)		55 (8.0%)		17 (5.2%)		0.1431
Normal body weight (*n* = 714)	714 (70.1%)		484 (70.1%)		230 (69.9%)		
Overweight (*n* = 148)	148 (14.5%)		91 (13.2%)		57 (17.3%)		
Obesity (*n* = 85)	85 (8.3%)		60 (8.7%)		25 (7.6%)		

* non-normal distribution (Shapiro–Wilk test; *p* < 0.05); ** for individuals aged ≥18 years; *** for individuals aged <18 years; BMI—Body Mass Index; SD—standard deviation; IQR—Interquartile range.

**Table 2:** Comparison of Food Behavior Checklist (FBC) subscale scores for the adolescents studied in the PLACE-19 Study between the post-pandemic and pandemic periods at the one-year interval.

Food Behavior Item	Post-Pandemic		Pandemic		*p* **	Effect Size ***
	Mean ± SD	Median (IQR)	Mean ± SD	Median * (IQR)		
Fruit and vegetable quantity subscale (Q6, Q7)	7.1 ± 2.8	7.0 (4.0)	6.8 ± 2.8	6.0 (3.0)	<0.001	0.294
Fruit and vegetable variety subscale (Q1, Q3, Q8, Q9, Q14)	10.9 ± 2.7	11.0 (4.0)	10.2 ± 2.7	10.0 (3.0)	<0.001	0.352
Sweetened beverage subscale (Q2, Q4)	5.7 ± 1.3	6.0 (2.0)	5.6 ± 1.4	6.0 (1.0)	0.011	0.128
Milk/dairy subscale (Q5, Q10)	3.9 ± 1.2	4.0 (2.0)	4.1 ± 1.2	4.0 (2.0)	<0.001	−0.375
Healthy fat subscale (Q11, Q12)	3.8 ± 1.4	4.0 (2.0)	3.9 ± 1.4	4.0 (2.0)	<0.001	−0.316
Diet quality subscale (Q13, Q16)	8.4 ± 2.3	8.0 (3.0)	8.4 ± 2.3	9.0 (3.0)	0.059	−0.078
Food security item (Q15)	3.8 ± 0.5	4.0 (0)	3.8 ± 0.6	4.0 (0)	0.002	0.365
Questions related to fruit and vegetable (Q1, Q3, Q6, Q7, Q8, Q9, Q14)	17.2 ± 4.8	18.0 (7.0)	17.1 ± 5.0	17.0 (6.0)	<0.001	0.353
Total scale (Q1–Q16)	43.6 ± 7.3	44.0 (10.0)	42.9 ± 7.4	43.0 (9.0)	0.020	0.091

* non-normal distribution (Shapiro–Wilk test; *p* < 0.05); ** Wilcoxon Signed-Rank test; *** Rank-biserial correlation (RBC); SD—Standard Deviation; IQR—Interquartile range.

**Table 3:** Comparison of Food Behavior Checklist (FBC) subscale scores for the female adolescents studied in the PLACE-19 Study between the post-pandemic and pandemic periods at the one-year interval (*n* = 690).

Food Behavior Item	Post-Pandemic		Pandemic		*p* **	Effect Size ***
	Mean ± SD	Median (IQR)	Mean ± SD	Median * (IQR)		
Fruit and vegetable quantity subscale (Q6, Q7)	7.3 ± 2.8	7.0 * (4.0)	7.0 ± 2.8	7.0 (4.0)	<0.001	0.289
Fruit and vegetable variety subscale (Q1, Q3, Q8, Q9, Q14)	11.1 ± 2.8	11.0 * (4.0)	10.4 ± 2.7	10.0 (3.0)	<0.001	0.307
Sweetened beverage subscale (Q2, Q4)	5.9 ± 1.3	6.0 * (2.0)	5.7 ± 1.4	6.0 (2.0)	<0.001	0.226
Milk/dairy subscale (Q5, Q10)	3.8 ± 1.2	4.0 * (2.0)	4.0 ± 1.2	4.0 (2.0)	<0.001	−0.415
Healthy fat subscale (Q11, Q12)	3.9 ± 1.4	4.0 * (2.0)	4.0 ± 1.4	4.0 (2.0)	<0.001	−0.299
Diet quality subscale (Q13, Q16)	8.2 ± 2.3	8.0 * (3.0)	8.2 ± 2.3	8.0 (3.0)	0.807	0.012
Food security item (Q15)	3.8 ± 0.5	4.0 (0)	3.8 ± 0.6	4.0 (0)	0.029	0.359
Questions related to fruit and vegetable (Q1, Q3, Q6, Q7, Q8, Q9, Q14)	18.4 ± 5.1	18.0 * (7.0)	17.4 ± 5.0	17.0 (7.0)	<0.001	0.307
Total scale (Q1–Q16)	44.0 ± 7.4	44.0 (10.0)	43.1 ± 7.5	43.0 (9.0)	0.020	0.109

* non-normal distribution (Shapiro–Wilk test; *p* < 0.05); ** Wilcoxon Signed-Rank test; *** Rank-biserial correlation (RBC); SD—Standard Deviation; IQR—Interquartile range.

**Table 4:** Comparison of Food Behavior Checklist (FBC) subscale scores for the male adolescents studied in the PLACE-19 Study between the post-pandemic and pandemic periods at the one-year interval (*n* = 329).

Food Behavior Item	Post-Pandemic		Pandemic		*p* **	Effect Size ***
	Mean ± SD	Median (IQR)	Mean ± SD	Median * (IQR)		
Fruit and vegetable quantity subscale (Q6, Q7)	6.7 ± 2.9	6.0 * (4.0)	6.5 ± 2.8	6.0 * (4.0)	0.004	0.303
Fruit and vegetable variety subscale (Q1, Q3, Q8, Q9, Q14)	10.5 ± 2.7	10.0 * (3.0)	9.9 ± 2.6	10.0 * (3.0)	<0.001	0.461
Sweetened beverage subscale (Q2, Q4)	5.4 ± 1.3	5.0 * (2.0)	5.4 ± 1.4	6.0 * (2.0)	0.316	−0.092
Milk/dairy subscale (Q5, Q10)	4.2 ± 1.2	4.0 * (2.0)	4.3 ± 1.2	4.0 * (1.0)	0.012	−0.263
Healthy fat subscale (Q11, Q12)	3.6 ± 1.3	3.0 * (3.0)	3.7 ± 1.1	4.0 * (1.0)	0.035	−0.158
Diet quality subscale (Q13, Q16)	8.7 ± 2.2	9.0 * (3.0)	9.0 ± 2.3	9.0 * (3.0)	<0.001	−0.289
Food security item (Q15)	3.9 ± 0.5	4.0 (0)	3.8 ± 0.5	4.0 (0)	0.024	0.387
Questions related to fruit and vegetable (Q1, Q3, Q6, Q7, Q8, Q9, Q14)	17.3 ± 5.1	17.0 * (7.0)	16.4 ± 5.0	16.0 * (6.0)	<0.001	0.473
Total scale (Q1–Q16)	42.9 ± 7.1	42.0 (10.0)	42.6 ± 7.2	42.0 (9.0)	0.116	0.077

* non-normal distribution (Shapiro-Wilk test; *p* < 0.05); ** Wilcoxon Signed-Rank test or paired-samples *t*-test; *** Rank-biserial correlation (RBC); SD—Standard Deviation; IQR—Interquartile range.

**Table 5:** Distribution of changes in total Food Behavior Checklist (FBC) score for the adolescents studied in the PLACE-19 Study from the pandemic to the post-pandemic period at the one-year interval, stratified by sex, body mass, age, and economic situation.

	Improved	Unchanged	Worsened	*p*
Total (*n* = 1019)	445 (43.7%)	163 (16.0%)	411 (40.3%)	-
**Sex**				
Female (*n* = 690)	310 (44.9%)	94 (13.6%)	286 (41.4%)	0.0114
Male (*n* = 329)	135 (41.0%)	69 (21.0%)	125 (38.0%)	
**Body mass**				
Underweight (*n* = 72)	25 (34.7%)	12 (16.7%)	35 (48.6%)	0.1653
Normal body weight (*n* = 714)	314 (44.0%)	104 (14.6%)	296 (41.5%)	
Overweight (*n* = 148)	66 (44.6%)	32 (21.6%)	50 (33.8%)	
Obesity (*n* = 85)	40 (47.1%)	15 (17.6%)	30 (35.3%)	
**Age**				
Minors (*n* = 692)	307 (44.4%)	108 (15.6%)	277 (40.0%)	0.7820
Young adults (*n* = 327)	138 (42.2%)	55 (16.8%)	134 (41.0%)	
**Economic situation**				
Poor/very poor (*n* = 26)	7 (26.9%)	4 (15.4%)	15 (57.7%)	0.1865
Average (*n* = 186)	71 (38.2%)	37 (19.9%)	78 (41.9%)	
Good (*n* = 529)	236 (44.6%)	84 (15.9%)	209 (39.5%)	
Very good (*n* = 278)	131 (47.1%)	38 (13.7%)	109 (39.2%)	

## Data Availability

The data presented in this study are available on request from the corresponding author due to ethical restrictions.

## Abbreviations

The following abbreviations are used in this manuscript:

BMI	Body Mass Index
CAWI	Computer-Assisted Web Interview
COVID-19	Coronavirus Disease
CSO	Central Statistical Office
FBC	Food Behavior Checklist
GSHS	Global School-Based Health Survey
HABITS	Health and Behavior in Teenagers Study
HBSC	Health Behaviour in School-aged Children
HEBECO	Health Behaviours During the COVID-19 Pandemic
IFAD	The International Fund for Agricultural Development
IQR	Interquartile range
NER	Net Enrollment Rate
PLACE-19	Polish Adolescents’ COVID-19 Experience Study
PMNCH	Partnership for Maternal Newborn and Child Health
RBC	Rank-Biserial Correlation
UNICEF	The United Nations Children’s Fund
WHO	World Health Organization
WFP	The World Food Programme
